#  The Effects of Extending of Co-planarity in a Series of Structurally Relative Polypyridyl Palladium(II) Complexes on DNA-binding and Cytotoxicity Properties 

**Published:** 2014

**Authors:** Somaye Shahraki, Hassan Mansouri-Torshizi, Ziba Sori Nezami, Arezou Ghahghaei, Fatemeh Yaghoubi, Adeleh Divsalar, Ali-Akbar Saboury, Farshad H. Shirazi

**Affiliations:** a Department of Chemistry, University of Sistan & Baluchestan, Zahedan, Iran.; b Department of Biology, University of Sistan & Baluchestan, Zahedan, Iran.; c Department of Biological Sciences, Tarbiat Moallem University, Tehran, Iran.; d Institute of Biochemistry and Biophysics, University of Tehran, Tehran, Iran.; e Pharmaceutical Sciences Research Center, Shahid Behesthi University of Medical Sciences, Tehran, Iran.

**Keywords:** Pd(II) complexes, Cytotoxicity, DNA binding

## Abstract

In depth interaction studies between calf thymus deoxyribonucleic acid (CT-DNA) and a series of four structurally relative palladium(II) complexes [Pd(en)(HB)](NO_3_)_2_ (a-d), where en is ethylenediamine and heterocyclic base (HB) is 2,2'-bipyridine (bpy, a); 1,10-phenanthroline (phen, b); dipyridoquinoxaline (dpq, c) and dipyridophenazine (dppz, d) (Figure 1), were performed. These studies have been investigated by utilizing the electronic absorption spectroscopy, fluorescence spectra and ethidium bromide (EBr) displacement and gel filtration techniques. a-d complexes cooperatively bind and denature the DNA at low concentrations. Their concentration at midpoint of transition, L_1/2_, follows the order a >> b > c > d. Also the g, the number of binding sites per 1000 nucleotides, follows the order a >> b ~ c > d. EBr and Scatchard experiments for a-d complexes suggest efficient intercalative binding affinity to CT-DNA giving the order: d > c > b > a. Several binding and thermodynamic parameters are also described. The biological activity of these cationic and water soluble palladium complexes were tested against chronic myelogenous leukemia cell line, K562. b, c and d complexes show cytotoxic concentration (Cc_50_) values much lower than cisplatin.

## Introduction

DNA is an interesting anionic polyelectrolyte plays a fundamental role in the storage and expression of genetic information in a cell. The interaction of DNA with cationic metal complexes containing various ligands has been an active area during the last thirty years (1-4). These complexes bind to DNA through a series of binding models, such as the electrostatic interaction of the cationic molecules with phosphate group of DNA, hydrogen-bounding, Vander Waals interaction of functionalities bond along the groove of the DNA helix and π-stacking interactions associated with intercalation of a planar aromatic group between the base pairs (5-7).

Metallointercalators are being used at the forefront of many of the agents that could bind and react with DNA (8). Increasing the surface area for intercalative stacking by a metal complex leads to a substantial increase in intercalative binding affinity. As a result, metallointercalator which contain an extended aromatic heterocyclic ligand can provide immensely powerful tools to probe nucleic acid (9-12). Metal complexes containing aromatic chelators are of great interest since they exhibit numerous biological properties such as antitumor, antibacterial and anticandidal activity (13-15). At the same time, metal complexes bearing ethylenediamine have also been interest because in the classical antitumor agent cisplatin, one of the ligands must be an N-donor and posses at least one hydrogen atom attached to the nitrogen (16). Moreover, a substantial investigation of the metals other than platinum (Ti, Ga, Ge, Pd, Au, Co and Sn) is underway that may help to avoid, or improves the problem associated with the use of platinum complexes as therapeutic agents (17,18).

**Figure 1:**
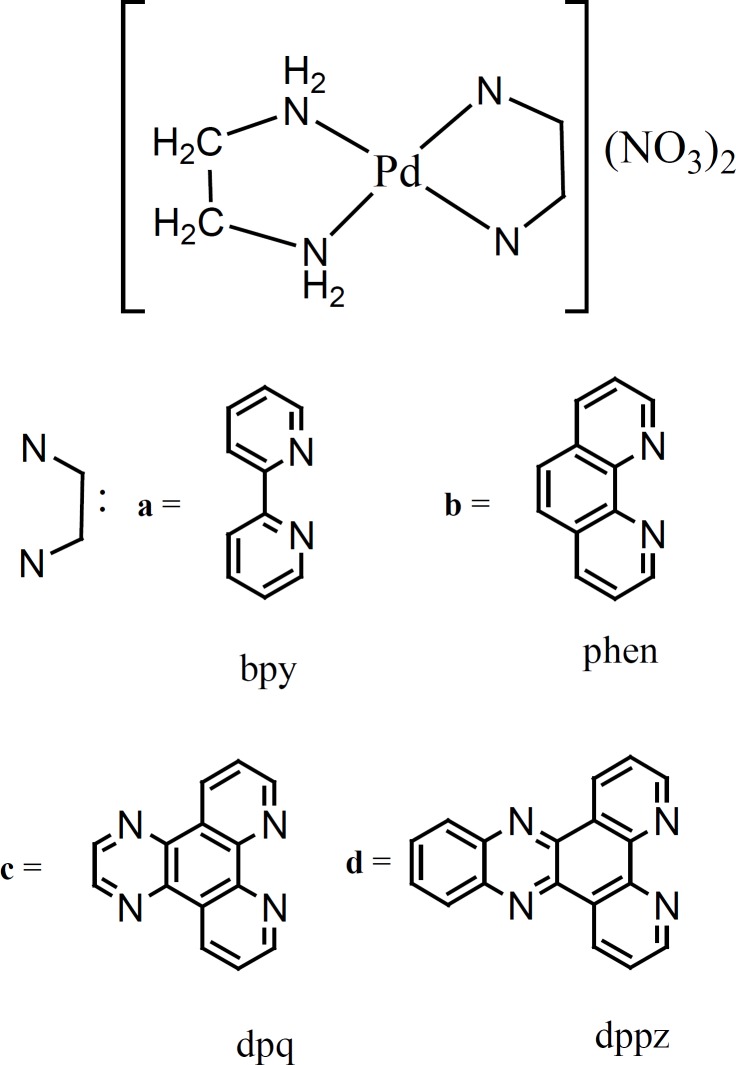
chemical structure of the complexes a-d

Herein we report in detailed CT-DNA-binding and cytotoxic studies of a series of four structurally relative palladium(II) square planar complexes. They contain aromatic moieties of two (bpy), three (phen), four (dpq) and five (dppz) rings (Figure 1). To the best of our knowledge no previous comparative DNA-binding studies for such a compounds are available in the literature. For this point of view the results presented here are of interest. A further reason of interest in these complexes is that, when the nonaromatic ligand (ethylenediamine, en), the metal center (Pd(II)), counter ion (NO_3_^-^) and experimental conditions for complexes in the series are the same, any difference in the thermodynamic-/binding-parameters and cytotoxicity data, must be due to stepwise ring addition to the aromatic moieties of the complexes for intercalation. Thus comparing the data become easy, straightforward, useful and conclusive.

## Experimental

*Material and Methods*

All reagents and solvents are of analytical reagent grade and doubly distilled water was used as solvent all along. The a-d complexes were prepared according to reported procedures in the literature (19,20). All the experiments involving interaction of the complexes with Calf thymus DNA (CT- DNA) were performed in Tris-HCl buffer (20mM) of pH =7.0 medium containing 20 mM NaCl. Ethidiumbromide (EBr) and CT-DNA were obtained from Sigma Chemical Co. (USA). Stock solution of DNA was prepared in Tris-HCl buffer and stored at 4˚C and its concentration was determined by UV absorbance at 260 nm using the molar extinction coefficient (ε) of 6600 M^−1^ cm^−1^. Solutions of CT-DNA in the buffer gave a ratio of UV–Vis absorbance of 1.8–1.9:1 at 260 and 280 nm, indicating that the DNA was sufficiently free of protein (21). Electronic absorption spectra of the title metal complexes were measured on a J_AS.CO _UV/Vis-7850 recording spectrophotometer. Fluorescence intensity measurements were carried out on a Varian spectroﬂuorimeter model Cary Eclips. 

*Anti-tumor studies*

The procedure for cytotoxic studies of a-d complexes were similar to the one reported earlier (22,23). Here also 1×10^4^ cells per ml of K562 Chronic myelogenous leukemia were used in Tris–HCl buffer solution of pH 7.0. In this experiment, the clear stock solution (2 mM, in deionized water) was sterilized by ﬁltering through sterilizing membrane (0.1 nm) and then varying concentrations of the sterilized drugs were added to harvested cells.

*Spectroscopic study*

*Denaturation of DNA*

This experiment was done by looking at the changes in the UV absorption spectrum of DNA solution at 260 nm upon addition of Palladium(II) complexes. Addition of metal complex to DNA solution was continued until no further changes in the absorption readings were observed. These absorption readings of DNA solutions were plotted separately versus different concentrations of metal complexes at two temperatures of 300 and 310 K. From these plots, the concentration of each metal complex at midpoint of transition, [L]_1/2_, for the two temperatures, could be deduced. Also, thermodynamic parameters such as: ${∆G}_{\left(\mathrm{H2O}\right)}^{{}^{\circ}}$, conformational stability of DNA in the absence of metal complex; ${∆H}_{\left(\mathrm{H2O}\right)}^{{}^{\circ}}$, the heat needed for DNA denaturation in the absence of metal complex; ${∆S}_{\left(\mathrm{H2O}\right)}^{{}^{\circ}}$, the entropy of DNA denaturation by metal complex as well as m, measure of the metal complex ability to destabilize DNA were found out using Pace method (24,25). 

*Spectral titration*

In the spectral titration two main experiments were carried out: (i) a fixed amount of each metal complex was titrated with increasing concentration of DNA. In this experiment, change in absorbance, ΔΑ, was calculated by subtracting the absorbance reading of mixed solutions of each metal complex with various concentrations of DNA, from absorbance reading of free metal complex. The maximum ΔΑ (ΔΑ_max_) of the metal complex totally bound to DNA was determined by interpolation of a plot of reciprocal of ΔΑs against the reciprocal of [DNA] obtained from each DNA concentration (i.e., intercept on ordinate). This ΔΑ_max_ was used to calculate the concentration of bound metal complex with DNA in the next experiment: (ii) a fixed amount of DNA was titrated with varying concentration of each metal complex. Here, also the ΔΑs for each concentration of metal complex were calculated as in experiment (i) at λ_max_ (nm) of each metal complex. In this experiment the concentration of Pd(II) complex bound to DNA, [L]_b_, and the concentration of free metal complex, [L]_f_, are calculated by using the relationship [L]_b_=ΔA[L]_f_/ΔA_max_. Here [L]_f_=[L]_t_–[L]_b_ where [L]_t_ is the maximum concentration of each metal complex added to saturate all the binding sites of DNA and ν is the ratio of the concentration of bound metal complex to total [DNA]. Using these data (ν, [L]_f_ ), and equation ν = [L]_b_ /[DNA] the Scatchard plots (ν /[L]_f_ vs. ν) were constructed for the interaction of each metal complex at the two temperatures 300 K and 310 K. The binding parameters: n, K, and g, where n is Hill coefficient, K is apparent binding constant and g is the number of binding sites per 1000 nucleotides of DNA were determined according to reported method (26). All measurements were performed separately at 300 K and 310 K and repeating three times for these complexes.

*Fluorescence Studies*

Ethidium bromide (EBr) is a trypanocidal dye displaying several biological properties (27,28). It has been observed that a very marked increase in the fluorescence of the dye occurs on binding with DNA (29). In the present work, this phenomenon will serve as a basis for investigation of the effect of our palladium (II) complexes on the fluorescence intensity of EBr binding to nucleic acid: At first, 60 µM DNA was added to 2 µM aqueous EBr solution in 2 mL Tris-HCl buffer of pH =7.0. The ﬂuorescence of EBr is enhanced about 50 fold on its intercalation between base pairs of DNA (30,31). This solution was titrated with varying concentration of each metal complex and the ﬂuorescence emission spectra of the intercalated ethidium bromide with increasing concentration of each Pd(II) complexes were recorded.

Second, a fixed amount of CT-DNA (60 µM) in the absence and presence of each metal complex was taken, incubated for 4h, titrated with increasing concentration of EBr (2,4,6,…,20 µM) and their ﬂuorescence emission intensity at 605 nm was measured. Thus by carrying different sets of DNA-metal complexes corresponding to different r_f_ values (r_f _is the ratio of the concentration of metal complex to DNA concentration), the number of EBr molecules intercalated to DNA (C_b_) was then calculated using Scatchard equation, C_b_ = I_t_ – I_0_/(ν-1)K, where I_0_ is the ﬂuorescence emission intensity of EBr alone, I_t_ is the ﬂuorescence emission intensity of EBr + DNA or EBr + DNA-metal complex, K is the slope of the plote of I_0_ versus C_0_ (where C_0_ is concentration of EBr added) and ν is the ratio of ﬂuorescence emission intensity of the bound and free EBr under the same condition of excitation wavelength, concentration, temperature, and solvent. By knowing C_b_, r was calculated, which is the ratio of bound EBr to total DNA concentration added and C, the concentration of free EBr. On plotting the r/C versus r, the binding isotherms were constructed and were represented as ﬂuorescence Scatchard plots. Several control experiments had been carried out. The above metal complexes by themselves do not show any ﬂuorescence. There was no interaction between metal complexes and EBr. In addition, the metal complexes did not quench the ﬂuorescence of EBr. 

*Gel ﬁltration*

A Sephadex G-25 column was equilibrated with 20 mmol/L Tris-HCl buffer of pH 7.0 in the presence of 10 mmol/L sodium chloride and each metal complex was incubated with CT-DNA for 6 h at 300 K then passed through it. Elution was done with the same buffer and each fraction of the column (2.5 mL) was monitored spectrophotometrically at λ_max_ (nm) for palladium(II) complexes(a :308 nm; b: 280 nm; c: 293 nm; and d: 280 nm) and at 260 nm for interacted DNA–metal complex, respectively (32). Gel chromatograms are obtained by plotting of absorbance readings at the two wavelengths versus column fractions in the same plot. In this plot, the two peaks obtained may resolve or not. The former indicates that the DNA is separated from the metal complex and the binding of DNA to metal complex is weak. However, if the two peaks are not resolved, it indicates that the DNA is not separated from the metal complex and the binding is strong.

## Results and discussion

A series of four water soluble and antitumor complexes, [Pd(en)(bpy)](NO_3_)_2_, [Pd(en)(phen)](NO_3_)_2_, [Pd(en)(dpq)](NO_3_)_2_ and [Pd(en)(dppz)](NO_3_)_2 _were prepared in our laboratory according to literature procedures (19,20). In the structure of these complexes only polypyridyl ligands (bpy, phen, dpq and dppz) varied. Thus they are structurally relative and contain planar aromatic moieties of two (bpy), three (phen), four (dpq) and five (dppz) rings (Figure 1), through which they may intercalate in DNA. Following studies were carried out to investigate the effects of extending planar aromatic moieties of the complexes on their DNA-binding affinities. In all of these experiments selected concentration ranges of metal complexes were bellow the concentrations needed to denature the CT-DNA (Table 1). These binding affinities were compared with their In Vitro cytotoxic activities.

*Anti-tumor studies*

**Figure 2:**
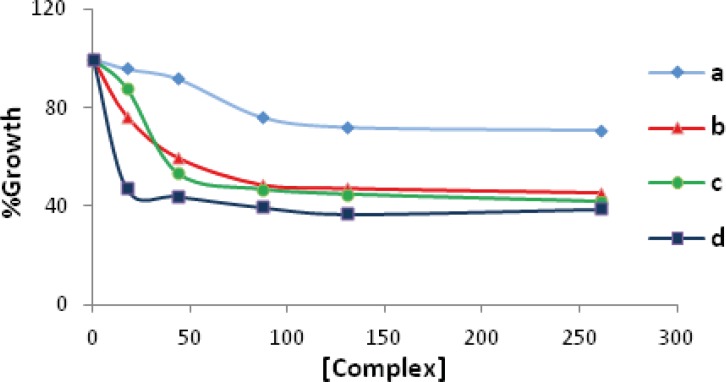
The growth suppression activity of the Pd(II) complexes **a**(♦) , **b** (▲), **c** (●) and **d** (■) on K562 cell line. The tumor cells were incubated with varying concentrations of the complexes for 24 h

The results of the cytotoxic activity on leukemia cell line K562 were determined according to the dose values of exposure of the complexes required to reduce survival of the cell lines to 50%. The 50% cytotoxic concentrations (Cc_50_) of Pd(II) complexes were determined 260.9 µM for a, 80 µM for b, 50 µM for c, and 16 µM for d complexes (see Figure 2). As shown in Figure 2, cell growing after 24 h was significantly reduced in the presence of various concentrations of the compounds. Furthermore, the Cc_50_ values of these complexes were compared to that found for anti-cancer agents used nowadays, that is, cisplatin under the same experimental conditions. This value (154 µM) is much higher as compared to b, c, and d complexes reported in this article.

*DNA-binding experiments*

*Denaturation study and* *determination of thermodynamic parameters*

**Figure 3:**
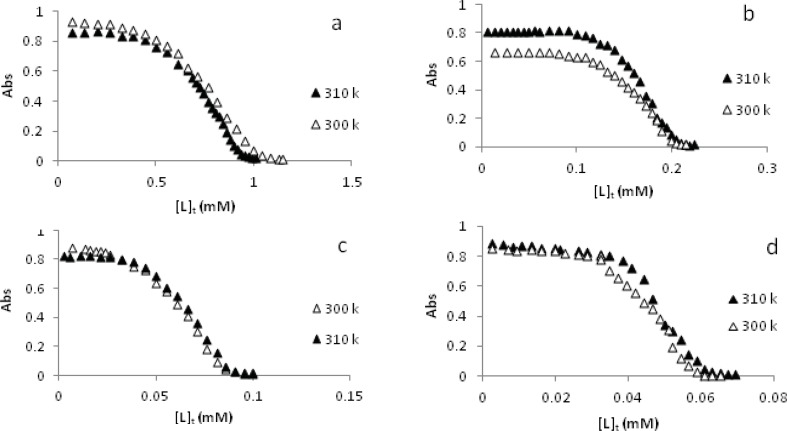
The changes of absorbance of CT-DNA at  _max_=260 nm due to increasing the concentration of [Pd(en)(bpy)](NO_3_)_2_ a, [Pd(en)(phen)](NO_3_)_2_ b, [Pd(en)(dpq)](NO_3_)_2_ c and [Pd(en)(dppz)](NO_3_)_2_ d complexes, at constant temperatures of 300 K and 310 K.

The above Palladium(II) complexes can denature DNA. The profiles of denaturation of DNA by a-d complexes at 300 and 310 K and the values of L_1/2 _so obtained are shown in Figure 3 and Table 2. Here we have observed two important points: (i) low values of [L]_1/2_ for these complexes, in particular c and d. This means that if these complexes will be used as anticancer agents, quite low doses will be needed, which may have fewer side effects. (ii) The [L]_1/2_ values decreases from a to d (see Table 2) which is in agreement with ring addition to the aromatic moieties of these complexes. Thus more the number of aromatic rings, more are the denaturing power of the complexes. These results are in alignment with results of cytotoxic studies and are comparable with [L]_1/2_ values of reported palladium(II) complexes (33-35). It is of note that the absorbance of CT-DNA base pairs (purines and pyrimidines) should increased by addition of a denaturing agent. However, the observed decrease in the absorbance of CT-DNA with increasing the concentration of each Pd(II) complex may be due to: (i) a possibility that interaction between CT-DNA and each metal complex causes the double helix of CT-DNA to become more straight leading to stacking. This stacking may cause conformational changes leading to a sort of denaturation, or (ii) each strand after denaturation gets associated in a more stacked structure and (iii) metal complex slips into the helix and masks the hydrophobic bases leading to a decrease in absorbance. As will be seen in the later part of this paper, the a-d complexes can bind CT-DNA taking the mode of intercalation. This mode of binding supports the above three hypothesis too.

**Figure 4:**
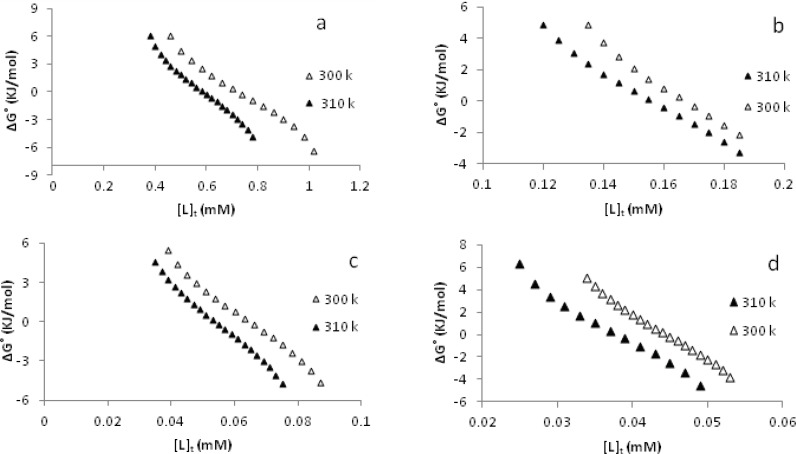
The molar Gibbs free energies plots of unfolding (∆G˚ vs [L]_t_) of CT-DNA in the presence of [Pd(en)(bpy)](NO_3_)_2_ a, [Pd(en)(phen)](NO_3_)_2_ b, [Pd(en)(dpq)](NO_3_)_2_ c and [Pd(en)(dppz)](NO_3_)_2_ d

Furthermore, some thermodynamic parameters found in the process of CT-DNA denaturation are discussed here: using the CT-DNA denaturation plots (Figure 3) and Pace method (24,25), the values of K, unfolding equilibrium constant and ∆G˚, unfolding free energy of DNA at two temperatures of 300 and 310 K in the presence of a-d complexes have been calculated. A straight line is observed when the values of ∆G˚ are plotted against the concentration of each metal complex in the transition region at 300 K and 310 K. These plots are shown in Figure 4. The values of m, that is the slope of these plots (a measure of the metal complexes ability to denature DNA) are in the order d > c > b > a (Table 2) suggesting the same order of number of rings in the aromatic moieties of the complexes. These values are comparable with those of Pd(II) complexes reported earlier (33-35). Furthermore, the intercept on ordinate ${∆G}_{\left(\mathrm{H2O}\right)}^{{}^{\circ}}$ (Figure 4) (conformational stability of DNA in the absence of metal complexes) are summarized in Table 2. As we know, the higher the values of ${∆G}_{\left(\mathrm{H2O}\right)}^{{}^{\circ}}$, the larger the conformational stability of CT-DNA. However, the values of ${∆G}_{\left(\mathrm{H2O}\right)}^{{}^{\circ}}$are decreased by rising the temperature. This is as expected because in general, the decrease in ${∆G}_{\left(\mathrm{H2O}\right)}^{{}^{\circ}}$ value is the main reason for the decrease in DNA stability (36). 

**Figure 5:**
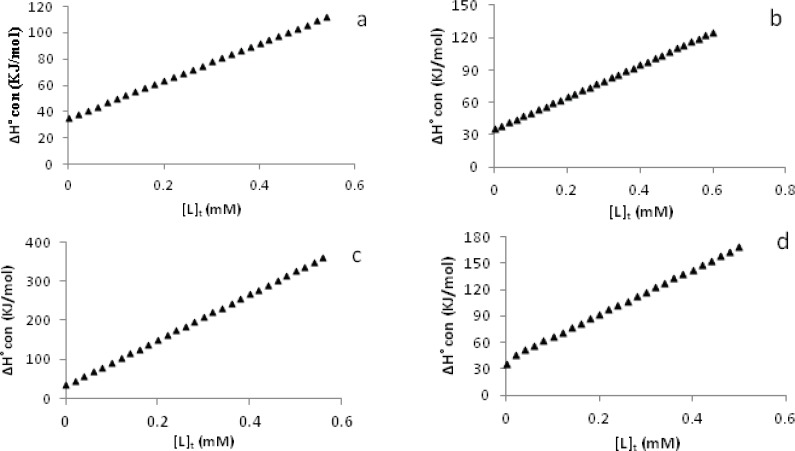
Plots of the molar enthalpies of DNA denaturation in the interaction with [Pd(en)(bpy)](NO_3_)_2_ a, [Pd(en)(phen)](NO_3_)_2_ b, [Pd(en)(dpq)](NO_3_)_2_ c and [Pd(en)(dppz)](NO_3_)_2_ d complexes, in the range of 300 to 310 K

 Another important thermodynamic parameter found is the molar enthalpy of DNA denaturation in absence of metal complexes i.e. ${∆H}_{\left(\mathrm{H2O}\right)}^{{}^{\circ}}$. For this, we calculated the molar enthalpy of DNA denaturation in presence of each metal complex, ΔH˚_conformation_ or ΔH˚_denaturation_, (ΔH˚_con_), in the range of the two temperatures using Gibbs-Helmholtz equation (37). In addition, the molar enthalpies of DNA denaturation in the absence of metal complexes, (${∆H}_{\left(\mathrm{H2O}\right)}^{{}^{\circ}}$), were determined by interpolation of a plot of ΔH˚ against the concentration of each metal complex. Straight lines will be obtained which are shown in Figure 5. Interpolation of these lines (intercept on ordinate i.e. absence of metal complex) give the values of ${∆H}_{\left(\mathrm{H2O}\right)}^{{}^{\circ}}$ (Table 2). These plots show that in the range of 300 to 310 K the changes in the enthalpies in the presence of Pd(II) complexes are ascending. These observations indicate that, on increasing the concentration of Pd(II) complexes, the stability of CT-DNA is increased. Also, the molar entropies of DNA denaturation, (${∆S}_{\left(\mathrm{H2O}\right)}^{{}^{\circ}}$), in the absence of each metal complex have been calculated using equation ΔG = ΔH–TΔS for each temperature (300 and 310 K) (Table 2). These data show that increasing temperature does not show concrete change in values of the entropies. This might be due to proximity of the temperature range. Also, the metal-DNA complexes are more disordered than the native DNA, because the entropy changes are positive (Table 2). These thermodynamic parameters compare favorably well with those of palladium (II) complexes as reported earlier (33-35). 

**Table 1:** Concentration ranges of metal complexes in the UV-vis/Fluorescence experiments[L]: concentration of each metal complex.

** [L]/[DNA]** **Gel Filtration Studies**	**[L]/[DNA]** **Fluorescence Studies**	**[L]/[DNA]** **UV-vis Studies**	**[L]/[DNA]** **Denaturation** ** Studies**	**Temperature (K)**	**Compound**
3.00	0.00-2.50	2.50-4.00	3.72-9.51 3.35-9.45	300 310	**[Pd(en)(bpy)](NO**_3_ **)**_2_
1.50	0.00-1.50	0.50-1.50	1.44-3.47 1.20-2.95	300 310	**[Pd(en)(phen)](NO**_3_ **)**_2_
0.80	0.00-1.00	0.30-1.00	0.85-1.97 0.79-2.03	300 310	**[Pd(en)(dpq)](NO**_3_ **)**_2_
0.50	0.00-0.50	0.20-0.65	0.55-1.05 0.51-1.10	300 310	**[Pd(en)(dppz)](NO**_3_ **)**_2_

**Table 2:** Thermodynamic parameters and values of L_1/2_ of DNA denaturation by palladium (II) complexes

**Compound**	**Temperature (K)**	a **L**_1/2_	b **m** **(kJ/mol)(mmol/L)** ^-1^	^c^ ${∆G}_{\left(\mathrm{H2O}\right)}^{{}^{\circ}}$ **(kJ/mol)**	^d^ ${∆H}_{\left(\mathrm{H2O}\right)}^{{}^{\circ}}$ **(kJ/mol)**	^e^ ${∆S}_{\left(\mathrm{H2O}\right)}^{{}^{\circ}}$ **(kJ/molK)**
**[Pd(en)(bpy)](NO**_3_ **)**_2_	300	0.66	18.89	15.56	35.54	0.067
	310	0.68	24.25	14.99		0.066
**[Pd(en)(phen)](NO**_3_ **)**_2_	300	0.154	131.9	15.56	35.39	0.066
	310	0.147	141.3	14.99		0.066
**[Pd(en)(dpq)](NO**_3_ **)**_2_	300	0.054	220.4	15.56	35.57	0.067
	310	0.053	247.3	14.99		0.066
**[Pd(en)(dppz)](NO**_3_ **)**_2_	300	0.046	385.5	15.56	35.81	0.067
	310	0.045	406.8	14.99		0.067

a The concentration of each metal complex at midpoint of transition

b Measure of the metal complex ability to denature CT-DNA.

c Conformational stability of CT-DNA in the absence of metal complex.

d The heat needed for CT-DNA denaturation in the absence of metal complex.

e The entropy of CT-DNA denaturation by metal complex.

*Electronic absorption titration and elucidation of DNA binding parameters*

**Figure 6:**
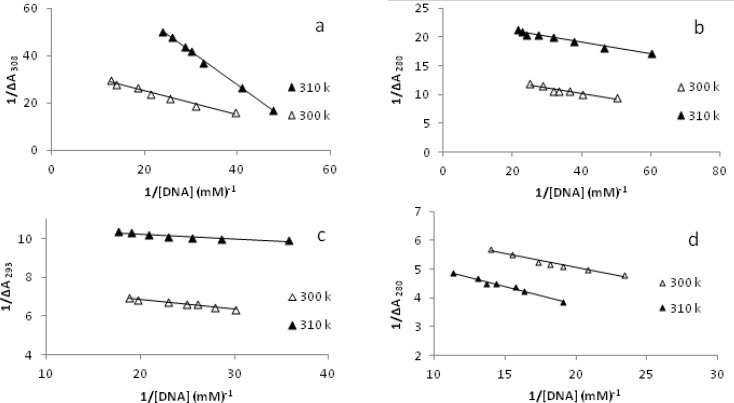
The changes in the absorbance of fixed amount of metal complexes in the interaction with varying amount of CT-DNA at 300 K and 310 K. The linear plot of the reciprocal of ∆A vs the reciprocal of [DNA] for [Pd(en)(bpy)](NO_3_)_2_ a, [Pd(en)(phen)](NO_3_)_2_ b, [Pd(en)(dpq)](NO_3_)_2_ c and [Pd(en)(dppz)](NO_3_)_2_ d complexes.

A fixed amount of each metal complex was titrated with increasing concentration of DNA in total volume of 2 mL at 300 K and 310 K, separately. In this experiment, change in absorbance, ΔA, was calculated by subtracting the absorbance reading of mixed solutions of each metal complex with various concentrations of DNA, from absorbance reading of free metal complex. The values of ΔA_max_, change in absorbance when all binding sites on DNA were occupied by each metal complex, are given in Table 3 and Figure 6.

**Figure 7:**
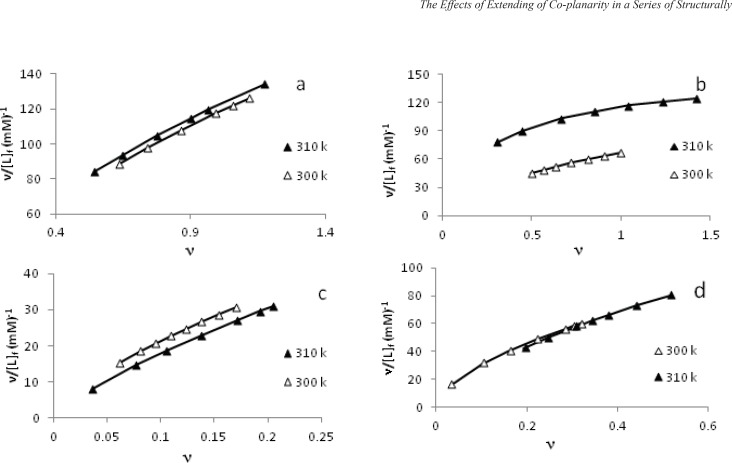
Scatchard plots for binding of [Pd(en)(bpy)](NO_3_)_2_ a, [Pd(en)(phen)](NO_3_)_2_ b, [Pd(en)(dpq)](NO_3_)_2_ c and [Pd(en)(dppz)](NO_3_)_2_ d, with CT-DNA

In another experiment, a fixed amount of DNA was titrated with varying amount of each metal complex. The concentration of each metal complex bound to DNA, [L]_b_, and the concentration of each free metal complex, [L]_f_ , are calculated by using the relationship [L]_b_=ΔA[L]_f_/ΔA_max_. Here [L]_f_ = [L]_t_ –[L]_b_ where [L]_t_ is the maximum concentration of each metal complex added to saturate all the binding sites of DNA and ν is the ratio of the concentration of bound metal complex to total [DNA]. Using these data (ν, [L]_f_ ), the Scatchard plots were constructed for the interaction of each metal complex at the two temperatures 300 K and 310 K. The Scatchard plots are shown in Figure 7 for a-d complexes. These plots are curvilinear concave downwards, suggesting cooperative binding (26).

**Table 3:** Values of ∆A_max_ and binding parameters in the Hill equation for interaction between CT-DNA and Pd(II) complexes in 20 mmol/L Tris-HCl buffer and pH 7.0.

**Compound**	**Temperature (K)**	a **∆A**_max_	b **g**	c **K (mol/L)** ^-1^	d **n**	e **Error**
**[Pd(en)(bpy)](NO**_3_ **)**_2_	300	0.028	7	0.019	8.50	0.0005
	310	0.011	7	0.023	8.03	0.0003
**[Pd(en)(phen)](NO**_3_ **)**_2_	300	0.071	5	0.039	2.64	0.003
	310	0.043	5	0.050	1.67	0.003
**[Pd(en)(dpq)](NO**_3_ **)**_2_	300	0.126	5	0.063	3.19	0.0006
	310	0.095	5	0.075	4.49	0.0003
**[Pd(en)(dppz)](NO**_3_ **)**_2_	300	0.158	3	0.080	2.51	0.0008
	310	0.144	3	0.095	3.18	0.0002

a Change in the absorbance when all the binding sites on CT-DNA were occupied by metal complex.

b The number of binding sites per 1000 nucleotides.

c The apparent binding constant.

d The Hill coeﬃcient (as a criterion of cooperativity).

e Maximum error between theoretical and experimental values of ν.

To obtain the binding parameters, the above experimental data (ν and [L]_f_) were substituted in Hill equation, [ν = g(K[L]_f_)^n^/(1+(K[L]_f_)^n^)], to get a series of equation with unknown parameters n, K and g (see Table 3) (38,39). Using Eureka software (40), the theoretical values of these parameters could be deduced. The maximum errors between experimental and theoretical values of ν are also shown in Table 3 which is quite low. The K, apparent binding constant increases in the order d > c > b > a; n, the Hill coefficient (n = 1 indicates noncooperative, n > 1 is cooperative and n < 1 shows anticooperative binding of DNA with metal complex), indicate cooperative binding of DNA with a-d complexes and g, the number of binding sites per thousand nucleotides for DNA follows the order: a > b ≈ c > d. (see Table 3). 

**Figure 8:**
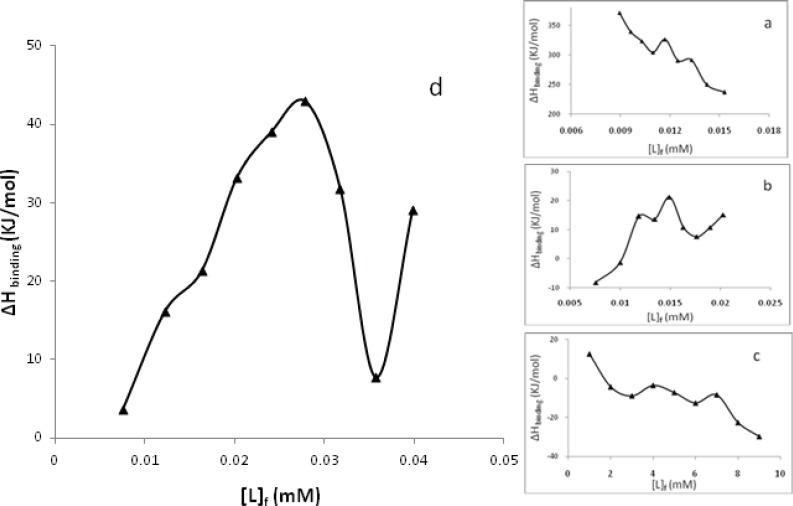
Molar enthalpies of binding in the interaction between CT-DNA and [Pd(en)(bpy)](NO_3_)_2_ **a**, [Pd(en)(phen)](NO_3_)_2_ **b**, [Pd(en)(dpq)](NO_3_)_2_ **c**, and [Pd(en)(dppz)](NO_3_)_2_ **d**, versus free concentration of complexes at pH 7.0 and 300 K

Also the experimental (dots) and theoretical (lines) values of ν in the Schatchard plots are super imposable on each other (Figure 7). Finding the area under plots of binding isotherms and using Wyman-Jons equation (24) we can calculate the K_app_ and ${∆G}_{\left(\mathrm{H2O}\right)}^{{}^{\circ}}$ at 300 and 310 K for each particular ν and also${∆H}_{\left(\mathrm{H2O}\right)}^{{}^{\circ}}$. Plots of the values of ${∆H}_{\left(\mathrm{H2O}\right)}^{{}^{\circ}}$ versus the values of [L]_f_ are shown in Figure 8 for the a-d complexes at 300 K. Deflections are observed in all plots. These deflections indicate that at particular [L]_f_, there is a sudden change in enthalpy of binding which may be due to binding of metal complexes to DNA or DNA denaturation. Similar observations can be seen in the literature where Pd(II) complexes have been interacted with CT-DNA (33-35). 

*Emission spectral studies and elucidation of the mode of binding*

**Figure 9:**
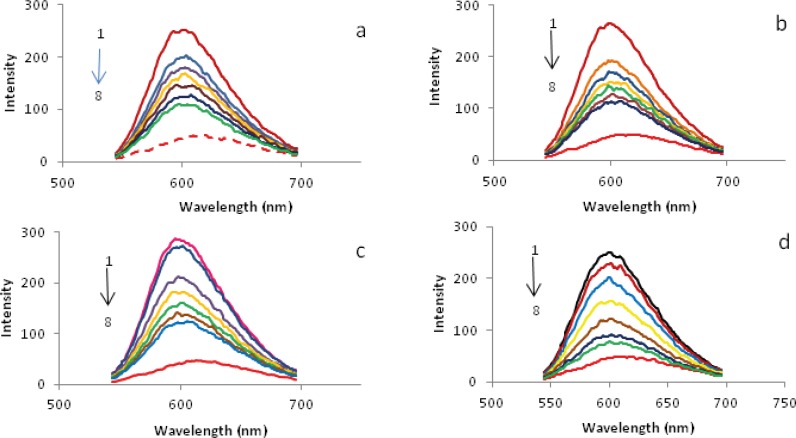
Florescence emission spectra of interacted EBr- CT-DNA in the absence (1) and presence (2-8) of different concentration of palladium(II) complexes:

It is interesting to note that the antitumour activity in vivo of palladium(II) comlexes is related to their mode of binding in vitro with DNA. The ﬂuorescence titration spectra have been conﬁrmed to be effective for characterizing the binding mode of the metal complexes to DNA (40). No ﬂuorescence was observed for the Pd (II) complexes either in aqueous solution or in the presence of CT-DNA. So the binding of above complexes with DNA cannot be directly presented in the emission spectra and thus have been studied by competitive ethidium bromide (EBr) binding experiments. In earlier literature, it was reported that the ﬂuorescent light of EBr–DNA system can be reduced by the addition of a second molecule (41), indicating the competition of second molecule with EBr in binding to DNA. The addition of Pd(II) complex caused the quenching fluorescence of the EBr-DNA system. This case can be considered as the complex directly reacted with the CT-DNA of DNA-EBr system, which leads to the EBr molecules left the EBr-DNA system, and the emission intensity of EBr-DNA system decreased (5). The emission spectra of EBr bound to CT-DNA in the absence and the presence of the Pd(II) complex are given in Figure 9. The addition of the complex to CT-DNA pretreated with EBr caused appreciable reduction in the emission intensity, indicating that the replacement of the EBr fluorophore by the complex results in a decrease of the binding constant of ethidium bromide to CT-DNA (35). 

[Pd(en)(bpy)](NO_3_)_2_ (**a**): 30µM(2), 50µM(3), 70µM(4), 90µM(5), 110µM(6), 130µM(7), EBr alone(8)

[Pd(en)(phen)](NO_3_)_2_ (**b**): 15µM(2), 30µM(3), 45µM(4), 60µM(5), 75µM(6), 90µM(7), EBr alone(8)

[Pd(en)(dpq)](NO_3_)_2_ (**c**): 10µM(2), 20µM(3), 30µM(4), 40µM(5), 50µM(6), 60µM(7), EBr alone(8) and

[Pd(en)(dppz)](NO_3_)_2_ (**d**): 5µM(2), 10µM(3), 15µM(4), 20µM(5), 25µM(6), 30µM(7), EBr alone(8)

**Figure 10:**
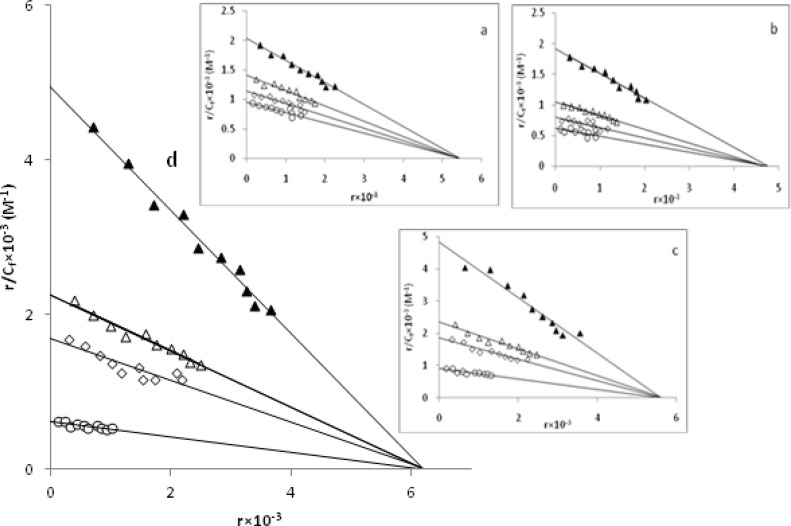
Competition between **a-d** complexes with ethidium bromide for the binding sites of CT-DNA (Scatchard plot). In curve 1 (▲), Scatchard's plot was obtained with calf thymus DNA alone that its concentration was 60µM. In curves nos. 2(∆), 3(◊) and 4(○) respectively 50µM, 100µM, 150µM of **a**, 30µM, 60µM, 90µM of **b**, 20µM, 40µM, 60µM of **c** and 10µM, 20µM, 30µM of **d **complexes at room temperature were added.

 Further studies to characterize the mode of binding of Pd(II) complexes to CT-DNA were carried out (24,35,43). The number of EBr molecules intercalated to CT-DNA in presence of different concentrations of the Pd(II) complex was calculated using Scatchard analysis (44). In this experiment, the wavelengths of excitation and emission were set at 540 nm and 700 nm respectively. Both have 0.5 nm slit widths. Solutions of CT-DNA, EBr and metal complexes were prepared in Tris-HCl buffer of pH 7.0. In this medium solutions of Pd(II) complexs were interacted with CT-DNA by incubating them at 300 and 310 K for 4 h, appropriate amount of EBr was then added to them and further incubated at room temperature (300 K) for 4 h and finally processed for fluorescence spectral measurement. Saturation curves of fluorescence intensity for Pd(II) complexes-DNA systems at different r_f_ values were obtained in the presence of increased concentrations of EBr (2, 4 to …, 20 µM). The fluorescence Scatchard plots obtained for binding of EBr to CT-DNA in absence (▲) and presence (∆, ◊, ○) of various concentrations of a-d complexes were shown in Figure. 10. This figure shows that these complexes inhibit competitively the EBr binding to CT-DNA (type-A behavior) (43), where number of binding sites n, (intercept on the abscissa) remain constant and the slope of the graphs, that is K_app_, (apparent association constant) decreases with increasing the concentration of Pd(II) complexes (Table 4). This implies that all of a-d complexes are intercalating in CT-DNA and thereby competing for intercalation sites occupied by EBr. The values of K_app_ and n, suggest that d is the best intercalator and for the others it varied as c > b > a. Compare their K_app_ values with those of other known CT-DNA-intercalative complexes which possess analogical structure; the Pd(II) complexes in our paper have similar or stronger aﬃnities with CT-DNA (34). 

**Table 4:** Binding parameters for palladium(II) complexes on the fluorescence of EBr in the presence of CT-DNA

**Compound**	a **r**_f_	b **K×10** ^5^ ** (M)** ^-1^	c **n**
**[Pd(en)(bpy)](NO**_3_ **)**_2_	0.00 0.83 1.66 2.5	0.372 0.259 0.209 0.175	0.0054
**[Pd(en)(phen)](NO**_3_ **)**_2_	0.00 0.50 1.00 1.50	0.401 0.223 0.168 0.130	0.0048
**[Pd(en)(dpq)](NO**_3_ **)**_2_	0.00 0.33 0.66 1.00	0.859 0.414 0.330 0.160	0.0056
**[Pd(en)(dppz)](NO**_3_ **)**_2_	0.00 0.16 0.33 0.50	0.795 0.362 0.268 0.100	0.0062

a Formal ratio of metal complex to nucleotide concentration.

b Association constant.

c Number of binding sites (n ) per nucleotide.

*Gel ﬁltration studies*

**Figure 11:**
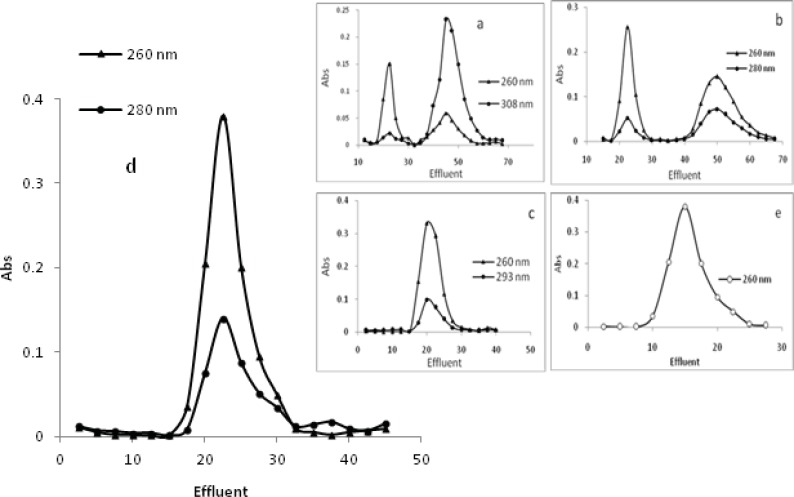
Gel chromatograms of intercalated CT-DNA with [Pd(en)(bpy)](NO_3_)_2_ **a**, [Pd(en)(phen)](NO_3_)_2_ **b**, [Pd(en)(dpq)](NO_3_)_2_ **c**, [Pd(en)(dppz)](NO_3_)_2_ **d **complexes and CT-DNA alone (e) obtained on Sephadex G-25 column, equilibrated with 20 mmol/L Tris-HCl buffer of pH 7.0 in the presence of 20 mmol/L sodium chloride

Results from the absorption and ﬂuorescence spectral studies show that the a-d complexes bind strongly to DNA. Binding of these complexes with DNA were also studied by gel filtration using a Sephadex G-25 column. CT-DNA solutions move on above gel and this movement is accelerated when they are bound to other molecules. Thus CT-DNA was interacted with a-d complexes in Tris-HCl buffer and then passed through a Sephadex G-25 column equilibrated with the same buffer. Elution was done with buffer and each fraction of the column was monitored spectrophotometrically. The gel chromatograms obtained from these experiments are given in Figure 11. The chromatograms of a and b show that two peaks were obtained for both aforementioned wavelengths and none of them were resolved. This indicates that in the presence of a and b complexes, CT-DNA partially breaks into two fragments, one with higher and the other one with lower molecular weight. These two Pd(II) complexes bound more to the fraction with higher molecular weight as is clear from the absorption reading at 308 nm for a and 280 nm for b. To confirm the breaking of CT-DNA by these metal complexes, a solution of CT-DNA alone was passed through the same column and each eluted fraction of 2 ml was monitored at 260 nm. The gel chromatogram obtained is shown in Figure 11 (e). This indicates that CT-DNA has fragments with approximately similar molecular weights.

 The plots of c and d complexes show that the peaks obtained for the two wavelengths are not resolved and suggest that CT-DNA has not separated from the metal complexes and their binding is sufficiently strong. This is due to the fact that if the interaction between CT-DNA and metal complexes was weak, the CT- DNA should have come out of the column separately (33).

## Conclusions

 Detailed analysis of the binding of four palladium(II) complexes having ethylenediamine and different heterocyclic diimine bases in a N_2_PdN_2_ square planar coordination geometry with CT-DNA by fluorescence, UV-Vis techniques and gel chromatography method are carried out in this work. In these complexes, stepwise ring addition to the aromatic moieties was employed. This planarity and extended conjugation of the heterocyclic bases have a profound effect on the DNA-binding and cytotoxic activity of a-d complexes. 50% cytotoxic concentration (Cc_50_) values of these complexes follows the order a >> b > c > d. Experimental results indicate that all complexes can cooperatively intercalate between the base pairs of DNA and their intercalation affinity to calf thymus DNA follows the order d > c > b > a which is in favor of ring addition to the aromatic moieties of the complexes. Several binding- and thermodynamic-parameters are also presented. Apparent binding constant, K_app_, obtained from UV-visible spectroscopic studies at two temperatures 300 and 310 K follows the order a > b > c > d. The same trends were observed for their concentration at midpoint of transition, L_1/2_, and g, the number of binding sites per 1000 nucleotides. Thus, almost all of the binding and thermodynamic parameters are in alignment with the results of cytotoxicity and number of rings present in the aromatic moieties of the complexes in the series. The bpy and phen complexes display breaking of DNA into two fragments and interact with both. 
